# Identification of metal species by ESI-MS/MS through release of free metals from the corresponding metal-ligand complexes

**DOI:** 10.1038/srep26785

**Published:** 2016-05-31

**Authors:** Munkhtsetseg Tsednee, Yu-Chen Huang, Yet-Ran Chen, Kuo-Chen Yeh

**Affiliations:** 1 Agricultural Biotechnology Research Center, Academia Sinica, Taipei, 11529 Taiwan

## Abstract

Electrospray ionization-mass spectrometry (ESI-MS) is used to analyze metal species in a variety of samples. Here, we describe an application for identifying metal species by tandem mass spectrometry (ESI-MS/MS) with the release of free metals from the corresponding metal–ligand complexes. The MS/MS data were used to elucidate the possible fragmentation pathways of different metal–deoxymugineic acid (–DMA) and metal–nicotianamine (–NA) complexes and select the product ions with highest abundance that may be useful for quantitative multiple reaction monitoring. This method can be used for identifying different metal–ligand complexes, especially for metal species whose mass spectra peaks are clustered close together. Different metal–DMA/NA complexes were simultaneously identified under different physiological pH conditions with this method. We further demonstrated the application of the technique for different plant samples and with different MS instruments.

Transition metals such as iron (Fe), zinc (Zn), and copper (Cu) play vital roles in the metabolism of all living organisms^1^,^2^,^3^. Mechanistic studies of how metals are transported, stored and incorporated as cofactors^4^ in cells or organisms require a systematic approach to measure the metal content, identify the metal-binding metabolites, peptides and/or proteins, and analyze the existence of metal–ligand complexes in the environment^5^. Analytical challenges often occur in metal speciation studies in biological samples. Such challenges are generally caused by the relatively low amounts and poor stability of the metal complexes during sample preparation and further analysis^6^.

X-ray absorption spectroscopy (XAS) can be used for direct analysis of the metal species in samples, with the surrounding metal-coordination environment, without a sample extraction step^7^,^8^,^9^. However, the requirement for a high metal concentration in the samples limits the general use of this method^5^. Therefore, the identification of metal complexes in a wide range of samples has mainly involved electrospray ionization-mass spectrometry (ESI-MS) because of its high selectivity and sensitivity along with gentle transition from the solution to gas phase^10^,^11^. ESI-MS generally generates singly charged metal-ligand spectra that can be identified with relative abundance of isotopic spectra corresponding to the naturally occurring metals. These spectra are called metal-specific isotopic spectra and/or isotopic patterns^5^,^12^. Furthermore, ESI-MS instruments allow for optimization of efficient detection conditions (*e.g.,* positive and negative ionization mode) for the metal complexes of interest across a range of solvents and pH conditions^5^,^6^. This approach can be extended with prior separation steps such as coupled liquid chromatography-MS (LC-MS)^13^ and capillary electrophoresis-MS (CE-MS)^14^, which further increase the detection sensitivity of different metal–ligand complexes^5^,^15^. In complex biological samples, particularly, the prior separation of metallo-metabolites (*e.g.*, using LC with a hydrophilic interaction chromatography column [HILIC])^16^,^17^ increases the detection of the target metal–ligand complexes by avoiding the signal suppression effects in complex mass spectra. However, the perturbations of the metal complex nature during the ionization process and accurately quantifying metal species in the analyte are still challenging with ESI-MS^6^,^11^.

Mass fragmentation of metal containing biomolecules such as platinum, lanthanide and iodine-containing peptides generated by high-energy collisional dissociation (HCD) was recently demonstrated as a promising application of MS to obtain molecular and elemental information in a single analysis^18^. The generated elemental ions in different MS instruments such as matrix-assisted laser desorption ionization (MALDI) imaging showed the potential use of this approach in metal speciation studies^18^.

Analysis of natural metal chelators such as the phytosiderophores (PSs) and their precursor nicotianamine (NA) and their metal complexes in plant samples is of particular interest because of their critical involvement in the acquisition and translocation of different nutrients in crops^6^,^19^,^20^. Metal–PSs and metal–NA complexes have been identified in several plant species by ESI-time-of-flight (TOF)-MS^21^ and LC-ESI-TOF-MS^22^,^23^ based on the isotopic signatures of the complexed metals.

In this study, we present tandem mass spectrometry (ESI-MS/MS) as an accurate and sensitive analytical procedure for identifying metal species. The method allows for identifying metal species by the release of free metals from the corresponding metal complexes. We illustrate the analytical procedures by analysis of different metal-deoxymugineic acid (DMA)/NA complexes as examples. The procedure may be applied in studies of metal species in a wide range of biological samples.

## Results

### ESI-MS/MS identification of metal species by release of free metals from metal–ligand complexes

To date, the identification of metal species with ESI-MS has relied directly on metal-specific isotopic signatures or isotopic patterns of the metal complex spectra^21^,^22^. Here, we report a method for identifying metal–ligand complexes by using ESI-MS/MS. Henceforth, “MS1” and “MS2” will be used to denote the spectra of primary ESI-MS and secondary ESI-MS/MS, respectively. In metal complex studies, we used five transition metals, Fe, Cu, nickel (Ni), Zn and cobalt (Co). The exact atomic masses and abundance of their natural isotopes (available at www.ciaww.org) are shown in Table 1. Analysis of these metal complexes in high-resolution ion-trap MS involved prepared standard metal–DMA and metal–NA complexes in positive ESI mode.

**Figure 1:**
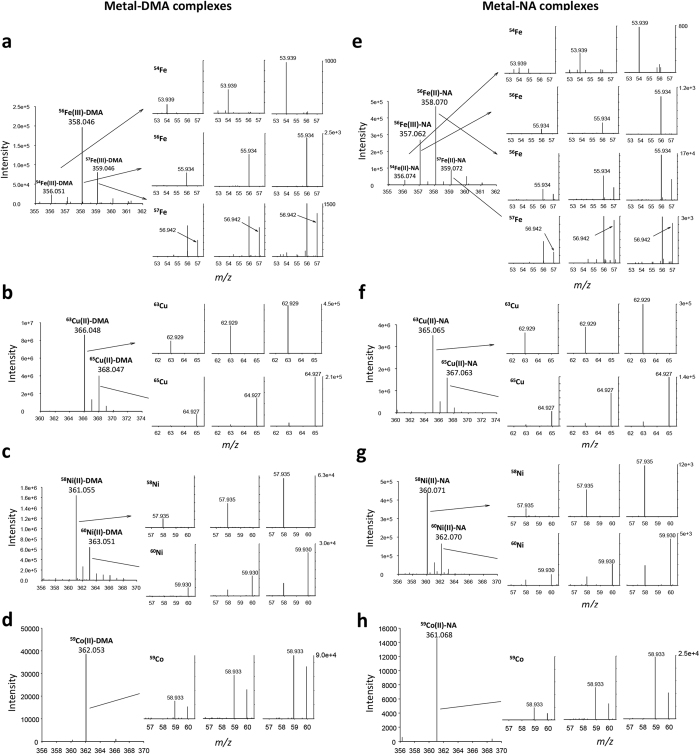
Release of free metals from metal–DMA and metal–NA complexes by ESI-MS/MS. The spectra of the released free metals (right panels) from the corresponding isotopic spectra of metal–DMA (**a–d**) and metal–NA (**e–h**) complexes were obtained in positive ESI mode with Orbitrap-MS at different fragmentation energies, HCD: 70–90%.

In MS1, metal–DMA/NA complexes can be identified with singly charged metal species in a metal–ligand stoichiometry of 1:1^21^,^22^. Formulations of the complexes were obtained as [M-H + Me(II)]^+^ and [M-2H + Me(III)]^+^ for di-valence and tri-valence metal–ligand complexes, respectively, as shown in the elemental composition in Table 2. Metal complexes were primarily identified in MS1 with high accuracy (<5 ppm), calculated from the comparisons of the calculated and observed *m/z* of metal–DMA/NA complexes (Table 2 and Supplementary Table S1). In MS1, the complex isotopic signatures also showed high accuracy, with <10% errors in relative isotopic abundance (RIA)^24^ (Table 2 and Supplementary Table S2). With this method, in MS2, during the fragmentations of isotopic spectra of metal complexes, we obtained product ion spectra that corresponded to the released free metals from the corresponding metal–DMA/NA complexes (Table 2 and Fig. 1). The identification of all the released free metals was confirmed with an exact atomic mass measurement of free metals and their isotopic signatures with high accuracy except for the low-abundant ^57^Fe isotope (Table 2 and Supplementary Table S3). Although the mass accuracy of free metal isotopes showed relatively low values (<10 and <14 ppm for major and minor isotopes, respectively) compared to those obtained in MS1, the values could still provide an accurate mass measurement^25^ in MS2. The measured mass accuracy increased under high fragmentation energies.

For Fe–DMA/NA complexes, we generated the product ions by fragmenting three isotopic Fe(III)–DMA complex spectra, *m/z* 356.051, 358.046 and 359.046, and three Fe(II)-NA complex spectra, *m/z* 356.074, 358.070 and 359.072, with different collision energies. We then acquired the product ion spectra for the release of free Fe isotopes, *m/z* 53.939, 55.934 and 56.942 for ^54^Fe, ^56^Fe and ^57^Fe, respectively, from their isotopic precursor spectra (Fig. 1a,e). The signal intensity of Fe spectra increased with increasing fragmentation energy applied to the precursors (HCD: 70–90%). Notably, the identification of the Fe(III)–NA complex in close isotopic spectra of the Fe(II)-NA complex was clearly separate from the release of ^56^Fe with *m/z* 55.934 from its precursor Fe(III)-NA, *m/z* 357.062 (Fig. 1e).

For Cu–DMA/NA complexes, we acquired the MS2 spectra from both isotopic precursors *m/z* 366.048 and 368.047 for the Cu(II)–DMA complex and *m/z* 365.065 and 367.063 for the Cu(II)–NA complex. The released free Cu isotopes, *m/z* 62.929 and 64.927 corresponding to ^63^Cu and ^65^Cu, from the corresponding isotopic parent spectra increased in abundance under different collision energies (Fig. 1b,f). For Ni–DMA/NA complexes, we generated the product ions from two isotopic precursors, *m/z* 361.055 and 363.051 for the Ni(II)–DMA complex and *m/z* 360.071 and 362.070 for the Ni(II)–NA complex. The product ion spectra for the released free Ni isotopes, *m/z* 57.935 and 59.930 for ^58^Ni and ^60^Ni, respectively, corresponded to the precursor spectra (Fig. 1c,g). For Co–DMA/NA complexes, we fragmented the parent spectra of *m/z* 362.053 and 361.068 for the Co(II)–DMA and Co(II)–NA complexes, respectively. The product ion spectrum identified as the released free ^59^Co single isotope was detected with *m/z* 58.933 in MS2 (Fig. 1d,h).

**Figure 2:**
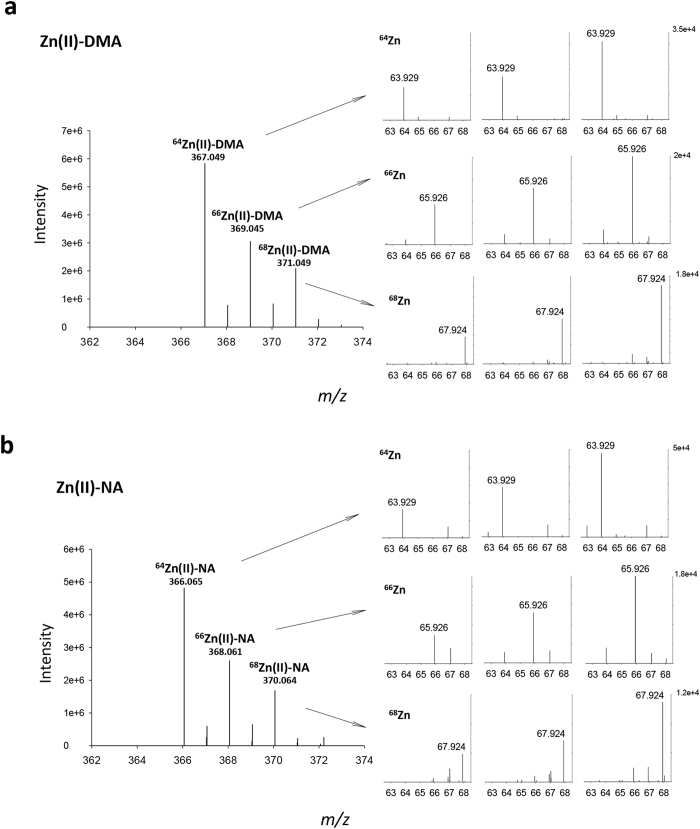
Spectra of the free Zn released from Zn(II)-DMA/NA complexes. The release of free Zn isotopes (right panels) from the corresponding Zn-complexes was obtained in positive ESI mode with Orbitrap-MS at fragmentation energy HCD 120, 130 and 150%.

For Zn–DMA/NA complexes, we acquired the product ions fragmented from three isotopic precursor ions, *m/z* 367.049, 369.045 and 371.049 for the Zn(II)–DMA complex and *m/z* 366.065, 368.061 and 370.064 for the Zn(II)–NA complex precursors, with high fragmentation energies. The released free Zn isotopes, ^64^Zn, ^66^Zn and ^68^Zn, were detected with *m/z* 63.929, 65.926 and 67.924, respectively in MS2 with corresponding isotopic parent ions (Fig. 2a,b). The detection sensitivities were lower for the free Zn isotopes than for other metals. We obtained Zn isotopic spectra by applying very high-energy collisional dissociation (HCD: 120–150%) with high concentrations of Zn-DMA/NA prepared solutions; other metal isotopes were generally detected at HCD: 50–90% with low concentrations of prepared solutions.

Of note, the released free metals from the metal complexes were detected only in positive but not negative ESI mode. Free metals were not released in MS2 spectra from free DMA and NA samples (Supplementary Figures S1 and S2).

Together, our data demonstrate a highly accurate method involving ESI-MS/MS for identifying metal species by the release of free metals from the metal–ligand complexes.

### Mass fragmentation analysis of metal–DMA and metal–NA complexes

**Figure 3:**
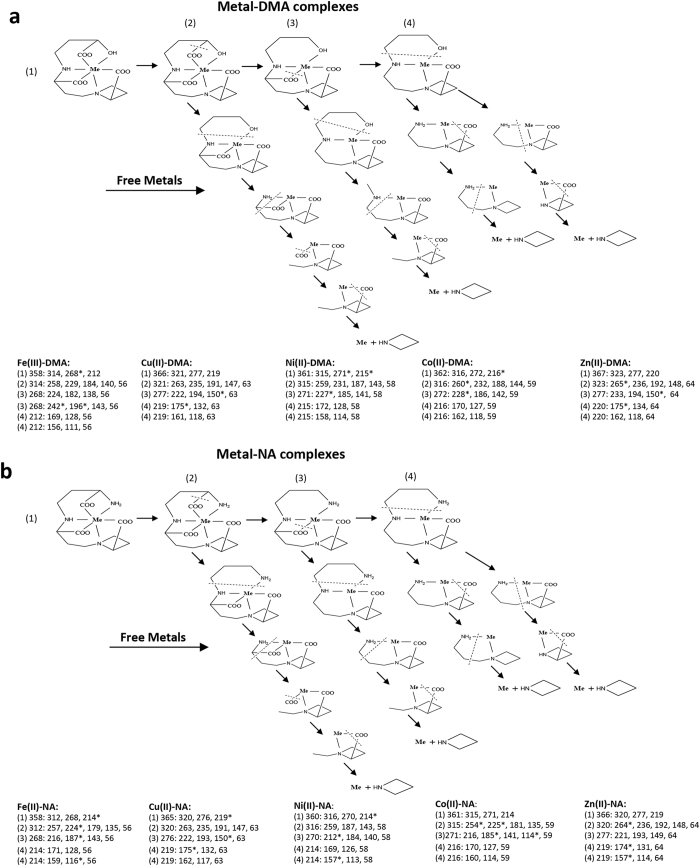
Proposed mass fragmentation pathways and product ions of metal–DMA and metal–NA complexes. Mass product ions (lower panels) of metal–DMA (**a**) and metal–NA complexes (**b**) were obtained in positive ESI mode with Obritrap-MS under different fragmentation energies, HCD: 40–90%. The proposed fragmentations and fragmentation pathways ((1)–(4)) are shown with dotted lines and arrows, respectively, accordingly to the *m/z* of product ions shown. “Free metals” on arrow indicates the fragmentations where the release of free metals was observed primarily. Asterisks denote the highest abundant fragments. “Me” in the structures represents the “Metal”.

Using the full spectra of MS/MS data from different metal–DMA/NA complexes obtained at different fragmentation energies (Supplementary Figures S3–S5), we proposed possible fragmentation pathways for metal–DMA (Fig. 3a) and metal–NA complexes (Fig. 3b). Despite slight differences in the protonation states of the fragmented product ions, the general fragmentation pathways of different metal–DMA and metal–NA complexes showed a similar pattern. Free metals were released from the metal–DMA/NA complexes in MS2 spectra with product ions generated at *m/z* ~150–200 (marked fragmentations in Fig. 3). We indicate the high-abundant product ions among the detected product ions for different metal–DMA/NA complexes in the proposed fragmentation pathways (Fig. 3, lower panels). The *m/z* of the neutral losses and the identification of lost fragments are in Supplementary Table S4.

In a similar way, mass fragmentation analysis of different metal–ligand complexes in *in vitro* and *in vivo* experiments may be useful for selecting product ions suitable for use in quantitative Multiple Reaction Monitoring (MRM)^26^ analysis and/or for analyzing the capacity of certain fragments that are essential for metal-binding and complex stability.

In addition, we tested the applied fragmentation energies that are required for releasing the free metals from the corresponding metal complex ions by using six different dilutions of Cu(II)–NA and Ni(II)–NA complex standard solutions. Results in MS2 spectra further showed the requirement for increased fragmentation energy for the released free Cu and Ni isotopes with decreased abundance of the Cu(II)–NA and Ni(II)–NA complexes in the series of diluted samples. In the same sample, the intensity of free Cu/Ni isotopes was increased under high collision energies, HCD: 80–90%, as compared with lower collision energies, HCD: 40–50%. The fragmentation energies also differed for different metal complexes because the Cu(II)–NA complex required lower percentages of collision dissociation energies than the Ni(II)–NA complexes (Table 3).

### Analysis of metal–DMA and metal–NA complexes in physiological pH conditions

**Figure 4:**
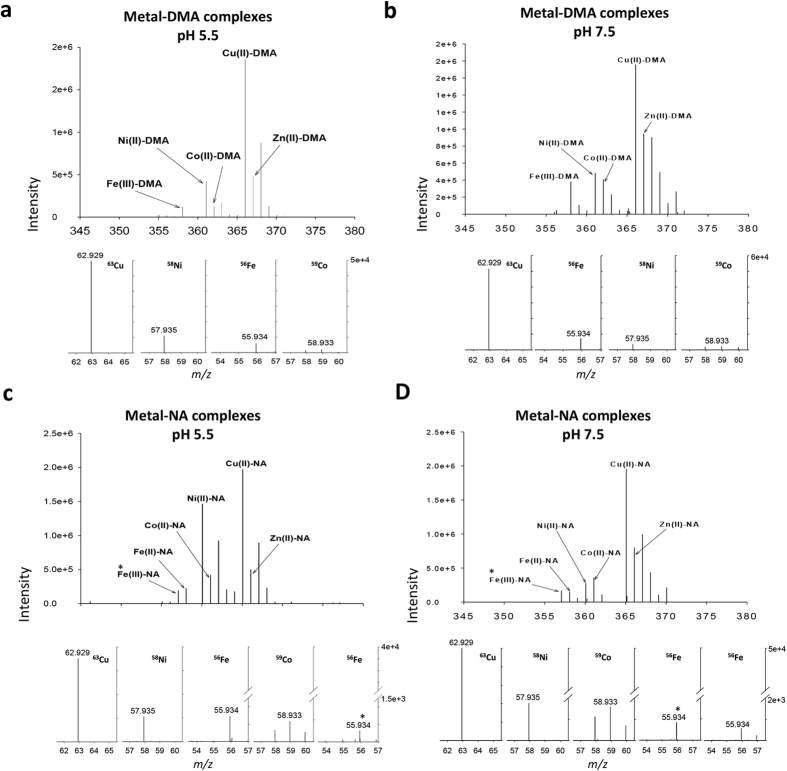
Identification of metal–DMA and metal–NA complexes under physiological pH conditions. The metal complex mixtures were prepared in pH 5.5 (**a,c**) and pH 7.5 (**b,d**) and identified with the released free metals (lower panel) in positive ESI mode with Obritrap-MS. The spectra of the released free metals were obtained in ESI-MS/MS with fragmentation energies HCD-90%. Asterisk denotes the ferric ion.

We next analyzed different metal–DMA/NA complexes simultaneously in one sample. The identification of metal complexes based on the metal-specific isotopic signatures is complicated in the presence of other metal complexes with peaks positioned close together, especially with low-abundance metal complexes in biological samples. In this analysis, we prepared a mixture of five different metal–DMA and metal–NA complexes. Compared to the isotopic spectra identified in Fig. 1 for separate metal complexes, with mixtures of different metal–DMA/NA complexes (Fig. 4, upper panels), the spectra of metal isotopic patterns were diminished by the other closely detected complex spectra. However, the use of MS2 with release of free metals from the metal complexes allowed for clear identification of the corresponding metal complex spectra. The abundance of the released free metals was also comparable to that of their precursor metal complex spectra (Fig. 4, lower panels).

We further analyzed the complex formations of different metals with DMA and NA under two physiological pH conditions, pH 5.5 and 7.5, as near-typical plant xylem and phloem pH values, respectively. In this *in vitro* experiment, different pH conditions were applied in background buffer to estimate the complexation nature of different metal-ligand complexes. The binding abilities of DMA and NA to different metals such as Fe, Zn, Cu, Ni, Co and manganese (Mn) were previously reported^16^,^21^ and the stabilities of the metal–NA complexes were also calculated^27^. However, the simultaneous identification of different metal–DMA and metal–NA complexes under physiological pH conditions to directly detect their dominant complex forms has not been described.

The direct infusion MS analysis we describe shows that among the identified metal–DMA complexes, DMA formed the most abundant complex with Cu(II) at both pH 5.5 and 7.5 (Fig. 4a,b). The relative abundances of the Cu(II)–DMA precursor complex and its released free Cu spectra were both higher than that of other metal–DMA complexes including Fe(III)–DMA. As noted previously, because of the poor detection sensitivity of the released free Zn spectra from Zn–DMA/NA complexes, we did not include it among the released free metals. The abundance of metal–DMA complexes did not change apparently under the two different pH conditions.

Similar to results obtained from the metal–DMA complexes, among the analyzed metal–NA complexes, the highest abundance was for Cu(II)–NA and its released free Cu spectra under the two pH conditions (Fig. 4c,d). The relative abundance of metal–NA complexes such as Fe(III)–NA, Cu(II)–NA and Ni(II)–NA varied slightly under the two different pH conditions. Cu(II)–DMA/NA complexes were also the most abundant forms under limited DMA and NA conditions (see Methods). Previously, the possible metal-exchange properties of NA were suggested during long-distance metal-NA transport in plants^6^. Thus, MS2 analysis of different metal–NA (and metal–DMA) complexes can be used to identify the different formed complexes in plant samples. Together, these data demonstrate the potential use of this method for the accurate and simultaneous identification of different metal complexes in a single sample.

### Application of the methodology in biological samples

**Figure 5:**
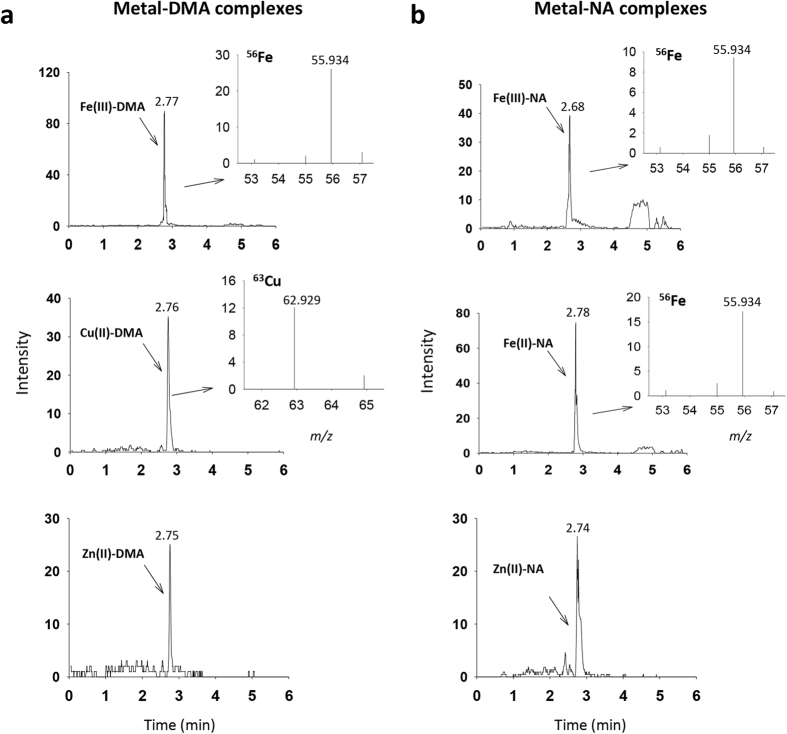
Identification of metal–DMA and metal–NA complexes in japonica rice shoots by ESI-MS/MS. Metal–DMA (**a**) and metal–NA (**b**) complexes in shoot extracts of japonica rice were identified in positive ESI mode with hydrophilic HILIC column separation by UPLC-ESI-Q-TOF-MS. The spectra of the released free metals were obtained in MS/MS with fragmentation energy 50 eV.

We used the method for analysis of a real sample of plant shoot extracts. We used a hydrophilic-interaction HILIC column before ESI-MS. With HILIC column separation in an Ultra Performance Liquid Chromatography (UPLC)-ESI-Q-TOF-MS instrument, we identified three metal–DMA complexes, Fe(III)–DMA, Cu(II)-DMA and Zn(II)–DMA, and three metal–NA complexes, Fe(III)–NA, Fe(II)–NA and Zn(II)–NA, in shoot extracts of japonica rice (Fig. 5a,b). Metal–DMA/NA complexes were first identified with the exact mass measurement on MS1 analysis (Table 1) and further confirmed in MS2 with the release of free metals from the corresponding metal complexes at fragmentation energy 50 eV. As described in Table 3, the relative abundance of the free metals released from the identified metal–DMA complexes appeared to differ at the same fragmentation energy. As well, under the conditions applied in MS2, the major isotopes were identified only from the free metals.

**Figure 6:**
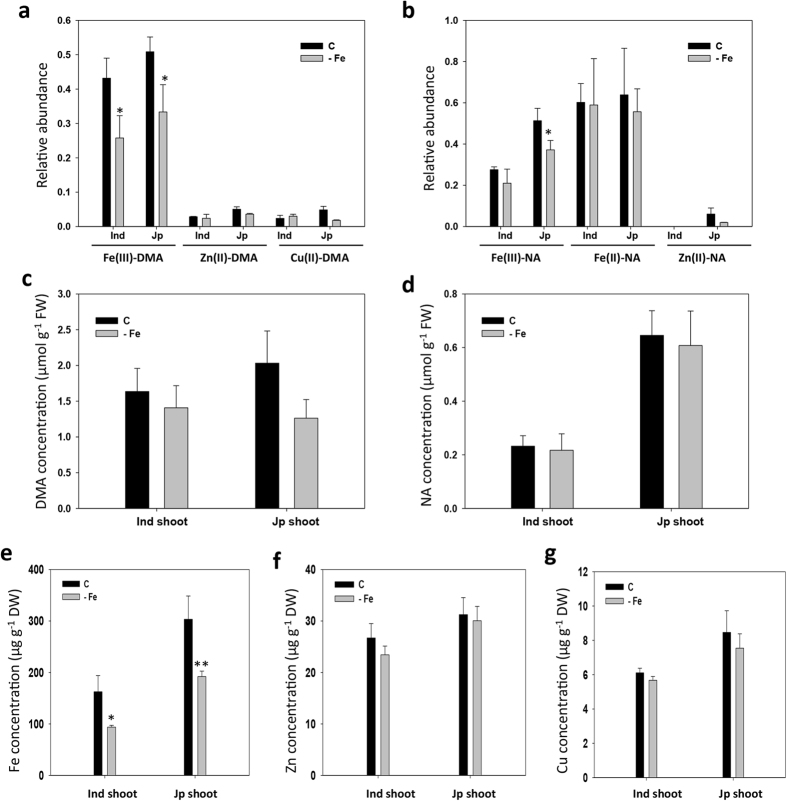
Concentrations of DMA and NA and nutrients in rice shoots. The relative abundance of the metal–DMA (**a**) and metal–NA (**b**) complexes in shoots of rice, indica (Ind) and japonica (Jp), was obtained by normalizing the peak area of the complexes to peak area of internal standard (leucine enkephalin, 2 ppm) in positive ESI mode by UPLC-ESI-Q-TOF-MS with a mean ± SD of three replicates. Shoot samples were collected from rice plants treated with control (C) and Fe deficiency (-Fe) for 7 days. The concentrations of DMA (**c**) and NA (**d**) in shoots samples were calculated in positive ESI mode by UPLC-ESI-Q-TOF-MS with a mean ± SD of three replicates. The concentrations of iron (**e**), zinc (**f**) and copper (**g**) in shoots of rice varieties were determined by ICP-OES. Data are the mean ± SD of three replicates with pooled 8 plants. Asterisks denote significant differences compared with control. ^*^P < 0.05; ^**^P < 0.01 by Student’s *t* test. FW, fresh biomass; DW, dry biomass.

To analyze the existence of different metal–DMA and metal–NA complexes in rice plants in response to Fe deficiency, we collected shoot samples from two rice varieties, indica and japonica, treated with Fe deficiency for 7 days. In the shoot of indica rice, we identified the same metal–DMA complexes as in japonica rice, and two metal–NA complexes, Fe(III)–NA and Fe(II)–NA (Supplementary Figure S6). In both rice varieties, Fe(III)–DMA and Fe(II)–NA complexes showed the highest abundance for DMA and NA chelators, respectively, with similar relative abundance (Fig. 6a,b). After their identification, the concentrations of free DMA and NA in the analyzed shoot samples were quantified by using authentic standards (Supplementary Figure S7). Both rice shoots showed lower concentrations of NA than DMA (Fig. 6c,d), which suggests the high stability of the Fe(II)–NA complex in these plants. We found a slight decrease in abundance of Fe(III)–DMA and Fe(III)–NA complexes under Fe deficiency, similar to the concentration of Fe in shoots (Fig. 6a,b,e). Compared with Fe complexes, the Cu(II)–DMA complex showed relatively low abundance (8 to 12 times lower). However, the concentration of Cu in shoots was significantly lower than that of Fe and Zn, especially 16 to 37 times lower than Fe content (Fig. 6e,f,g). Therefore, the data still suggest the possible high stability of Cu(II)–DMA complex in rice shoots.

Fe(III)–DMA, Cu(II)–DMA and Zn(II)–NA complexes were previously identified in tissues of rice^28^, barley^22^ and wheat^16^, with a role for DMA and NA suggested in the translocation of these nutrients in these grasses. In agreement with this suggestion, we identified the Cu(II)–DMA complex in addition to Fe(III)– and Zn(II)–DMA complexes in roots of wheat and barley plants with our method. Of note, in plant sample analysis, we used a different MS instrument (UPLC-ESI-Q-TOF-MS) rather than Orbitrap-MS to confirm the applicability of the method with different MS instruments. The data indicate the effective application of our method for biological samples and with different MS instruments.

## Discussion

In certain environments, the availability and reactivity of metal ions can be controlled by metal-binding ligands. Therefore, precise analysis of metal speciation is critical for studies related to metal biofunctions^6^,^29^. To date, analyses of metal species in a range of samples have generally involved ESI-MS^11^. However, the accurate identification of metal complex spectra to reveal metal ions was missing with this method. Here, we used MS/MS to detect product ion spectra that correspond to free metal release from five different precursor metal complexes formed by two natural organic ligands, DMA and NA.

We here present a multi-elemental approach compared with previously described MS/MS methods for the identification of NA, DMA and their metal complexes those mainly restrict to certain metals such as Fe^22^, Fe and Zn^28^, Ni^30^,^31^ and Cu^32^ in biological samples. Our method may offer two advantages over the traditional means of identifying metal species in MS1. In MS2, the relative abundance of free metal spectra increased with increasing fragmentation energies applied to the precursor spectra. This finding further indicates the potential use of the method to identify metal species in minute biological samples by applying high dissociation energies for fragmentation. Depending on the type of mass spectrometer used, fragmentation energies can be adjusted for target precursor ions^33^,^34^. The second advantage is the simultaneous identification of metal species in one sample. Because the method does not require a metal isotopic pattern in MS1, the identification of different metal complexes can be confirmed by the release of free metals from the precursor spectra. Simultaneous analysis of metal species in biological samples is particularly important to study the effect of one metal and/or one ligand on homeostasis of other metals^2^,^6^. However, an appropriate separation of different metal species with suitable chromatographic conditions in biological samples is still critical to avoid the mass interferences from isobaric co-eluted molecules.

In addition, mass fragmentation analysis such as MS2 and MS3 in MS/MS could be useful to study various metal-binding ligands including metabolites, proteins and enzymes to dissect their functional fragments and groups in the structures. Application of the method could also be extended in the screening of either targeted or untargeted metal complexes in a range of analytical samples and test platforms. Previously, the screening of isotopic pairs such as ^54^Fe-^56^Fe in LC-MS was used in the analysis of siderophores in culture extracts^35^,^36^. An open-source metal complex screening tool ChelomEx that identifies the isotope pattern-matched chromatographic peaks has recently been developed^37^. Our method could be useful to directly confirm the candidate species after such screening strategies for different metal complexes.

By using our method in *in vitro* experiments, we identified Cu(II)–DMA and Cu(II)–NA among other metal–DMA/NA complexes as major abundant complexes formed under two close physiological pH conditions. Analysis of metal complexes in shoots of two rice varieties further identified the Cu(II)–DMA complex in addition to Fe– and Zn–DMA/NA complexes in these plants. The relatively low content of Cu in the samples as compared with the Fe and Zn contents suggests the stable role of the Cu(II)–DMA complex that may be transported in vascular tissues. Previously, a study of the NA-free tomato *chloronerva* mutant reported the importance of NA for long-distance transport of Cu but not Fe^38^. *In vitro* analysis of Cu(II)–NA complex stability in a range of pH values has also suggested the role of NA in xylem transport of Cu(II)^21^. As well, Cu(II)–DMA was recently identified as the metal–DMA complex in rice xylem sap^32^.

In crops, NA and DMA are considered the crucial ligands for metal chelation^29^,^39^. Nearly half of the transgenic strategies used for biofortification in rice can manipulate the NA synthase gene (*NAS*) alone or with other genes^40^. Therefore, analyzing metal–DMA/NA complexes simultaneously in different tissues/parts of crops and transgenic plants would be of interest.

In conclusion, we have demonstrated an accurate method involving ESI-MS/MS for identifying metal species by the release of free metals from the corresponding metal–ligand complexes. This method could be useful with different MS instruments and different types of biological and environmental samples.

## Methods

### Chemicals

Deoxymugineic acid (95%) and nicotianamine (95%) were purchased from Toronto Research Chemicals (North York, Canada). Acetonitrile (analytical grade, Fluka), leucine enkephalin (LC-grade), ammonium bicarbonate (>99%), Ni(II)-nitrate (100%) and Co(II)-nitrate (100%) were from Sigma (St. Louis, MO, USA). Formic acid (analytical grade), methanol (LC-grade), Fe(III)/Fe(II)-chloride (99.2%) and Zn(II)-sulfate (100%) were from the Mallinckrodt Baker Institute (Phillipsburg, NJ, USA). Cu(II)-sulfate (100%) was purchased from Riedel-de Haen (Seelze, Germany) and ultrapure water was obtained from a Milli-Q-system (Merck Millipore, MA, USA).

### Preparation of metal–DMA/NA standard complexes

For developing the ESI-MS/MS method, the metal–DMA/NA standard complexes were obtained by incubating 100 μM DMA and/or NA solutions with 100 μM of different metal standard solutions in distilled deionized water in the dark at room temperature (25 °C) overnight. For Zn–DMA/NA complex analysis, 1000 μM DMA/NA and 1000 μM Zn standard solutions were used. For the simultaneous analysis of different metal–DMA/NA complexes, the metal complex mixtures were obtained by incubating 100 μM DMA and/or NA together with five different metal standard solutions (10 μM each metal solution) at two different pHs. For DMA/NA limited mixture, 100 μM of 5 metals was mixed with 100 μM of DMA or NA. In total, 20 mM aqueous ammonium acetate and 20 mM aqueous ammonium bicarbonate buffer solutions were used for pH 5.5 and 7.5, respectively^21^,^22^. The prepared standard samples were further microfiltered and subjected to MS after adding the internal standard, leucine enkephalin at 2.0 ppm final concentration.

### Plant materials and extraction of shoot metabolites

Two rice varieties, *Oryza sativa* L. var. *indica* (Indica rice, IR64) and *O. sativa* L. var. *japonica* (Japonica rice, TNG67) were germinated and grown hydroponically in half-strength Kimura B culture solution with the following composition: 0.18 mM (NH_4_)_2_SO_4_, 0.27 mM MgSO_4_.7H_2_O, 0.09 mM KH_2_PO_4_, 0.18 mM CaNO_3_.4H_2_O, 30 μM Fe(III)-citrate, 9 μM MnSO_4_.H_2_O, 46 μM H_3_BO_3_, 9 μM H_2_MoO_4_, 0.7 μM ZnSO_4_.7H_2_O and 0.3 μM CuSO_4_.5H_2_O. The pH of the nutrient solution was adjusted to 4.7 and renewed every 3 days. We transferred 14-day-old rice plants to treatments for 7 days. For Fe deficiency treatment, Fe(III)-citrate was omitted and 150 μM Ferrozine was added into the culture solution. For metabolite extraction, the shoots (approximately 100 mg fresh weight) of 3-week-old plants were homogenized and extracted with 1 mL of 80% methanol for 30 min under sonication at 25 °C. After centrifugation, the microfiltered shoot extracts were directly analyzed with LC-MS.

### LC-ESI-Orbitrap-MS analysis

Metal–ligand complex analysis in an ion trap MS instrument involved a high-resolution electrospray ionization (ESI)-coupled Orbitrap Elite Mass spectrometer (Thermo Scientific Orbitrap Elite MS). In direct infusion ESI-MS analysis, 10 μL analyte samples was injected into the instrument by loop (Waters) injection and delivered to the ionization source (Heated-ESI, HESI) with subsequent injection of 10 μL of 0.1% formic acid (FA) in 80% acetonitrile (ACN). The Orbitrap mass analyzer was operated in positive ionization mode with a source voltage 2.5 KV, capillary temperature 360 °C and source heater temperature 350 °C. Data were acquired at resolution 15,000 with a Fourier transform (FT)-MS instrument in a range of mass-to-charge ratios (*m/z*) of 50–990 and isolation width of 2 Thomson. Tandem mass spectra (MS/MS; MS2) acquired with high-energy collisional dissociation (HCD) were produced at different collision energies, 40–150%, and 2.0 ppm tolerance window. Data were analyzed by use of Thermo Scientific Xcalibur software (Thermo Fishers).

### LC-ESI-Q-TOF-MS analysis

LC-ESI-Q-TOF-MS involved use of Ultra Performance Liquid Chromatography (UPLC) with an electrospray ionization SYNAPT HDMS G1 hybrid quadrupole time-of-flight mass spectrometer (both from Waters, Millford, MA, USA). The SYNAPT HDMS G1 was operated in positive ionization mode with electrospray capillary voltage 3.0 kV. The cone and nitrogen desolvation gas flows were 50.0 and 500.0 L/h, respectively. Desolvation temperature was 250 °C. Mass data acquisitions were set at *m/z* 200–400 and 50–370 for MS1 and MS2 spectra analysis, respectively, in centroid data mode with scan time 0.2 s and tolerance widow 0.02 Dalton. The precursor isolation window was 3 Thomson and the collision energy of MS/MS analysis was set at 50.0 eV. A UPLC BEH HILIC column (1.7 μm, 2.1 × 100 mm, Waters) was used at 25 °C with elution gradients of the mobile phase, which consisted of 0.1% FA in 2% ACN (buffer A) and 0.1% FA in 100% ACN (buffer B). The gradient conditions were buffer A, 5.0–5.0%, 0–1.0 min; 5.0–50.0%, 1.0–2.0 min; 50.0–60.0%, 2.0–4.0 min; 60.0–60.0%, 4.0–5.0 min; 60.0–5.0%, 5.0–5.10 min and 5.0–5.0%, 5.10–7.0 min. The flow rate was 0.4 mL min^−1^. Data were analyzed with use of MassLynx 4.1 software. In the same analytical conditions, the concentrations of DMA and NA were calculated by using the normalized peak areas against the standard linear equation built from 8 different concentrations of DMA and NA standards in extraction buffer with r^2^ >0.99.

### ICP-OES analysis

Metal concentrations in rice tissues were analyzed by Inductively Coupled Plasma-Optical Emission Spectrometry (ICP-OES, OPTIMA 5300, Perkin-Elmer, Wellesley, MA, USA) as described^41^. Shoot samples were washed with CaCl_2_ and deionized water and dried at 70 °C for 3 days. Dried samples were digested with acid in a MarsExpress microwave digestion system (CEM, Matthews, NC, USA).

## Additional Information

**How to cite this article**: Tsednee, M. *et al.* Identification of metal species by ESI-MS/MS through release of free metals from the corresponding metal-ligand complexes. *Sci. Rep.* **6**, 26785; doi: 10.1038/srep26785 (2016).

## Supplementary Material

Supplementary Information

## Figures and Tables

**Table 1:** Natural isotope abundances of the analyzed metals.

Metals	Isotope abundances (%)
Fe	55.9349 (92); 53.9396 (6); 56.9354 (2)
Cu	62.9296 (69); 64.9278 (31)
Ni	57.9353 (68); 59.9308 (26)
Zn	63.9291 (48); 65.9260 (28); 67.9248 (19)
Co	58.9332 (100)

Relative abundance of the natural isotopes is shown in parentheses.

**Table 2:** Estimations of calculated and observed *m/z* of metal–DMA/NA complexes and their released free metal.

	Elemental composition		Complex identification			Complex isotopic signature				Released free metal			
	[M]	[M + H]^+^	calculated *m/z*	observed *m/z*	range of Δppm	metal isotope	calculated isotopic *m/z*	calculated intensity, %	range of RIA error, %	free metal	calculated *m/z*	observed *m/z*	range of Δppm
*Metal-DMA complexes*													
DMA	C_12_H_20_N_2_O_7_	C_12_H_21_N_2_O_7_											
Fe(III)-DMA	Fe(III)-C_12_H_17_N_2_O_7_	Fe(III)-C_12_H_18_N_2_O_7_	358.0463	358.0464	0.0–2.0	^56^Fe	358.0463	100.0		Fe	55.9349	55.9347	3.6–8.9
						^54^Fe	356.0510	6.3	0.1–1.0		53.9396	53.9389	5.6–13.0
						^57^Fe	359.0468	2.4	0.1–2.7		56.9354	56.9420	n.a.
Cu(II)-DMA	Cu(II)-C_12_H_18_N_2_O_7_	Cu(II)-C_12_H_19_N_2_O_7_	366.0488	366.0480	0.5–2.3	^63^Cu	366.0488	100.0		Cu	62.9296	62.9294	1.6–9.5
						^65^Cu	368.0470	44.6	0.6–2.7		64.9278	64.9274	1.5–7.7
Ni(II)-DMA	Ni(II)-C_12_H_18_N_2_O_7_	Ni(II)-C_12_H_19_N_2_O_7_	361.0546	361.0548	0.3–3.0	^58^Ni	361.0546	100.0		Ni	57.9353	57.9352	1.7–6.9
						^60^Ni	363.0500	38.2	0.1–2.3		59.9308	59.9304	3.3–11.7
Zn(II)-DMA	Zn(II)-C_12_H_18_N_2_O_7_	Zn(II)-C_12_H_19_N_2_O_7_	367.0484	367.0494	1.4–4.9	^64^Zn	367.0484	100.0		Zn	63.9291	63.9290	1.6–9.4
						^66^Zn	369.0453	57.4	1.1–5.0		65.9260	62.9259	1.5–13.7
						^68^Zn	371.0441	38.7	2.3–9.6		67.9248	67.9245	4.4–10.3
Co(II)-DMA	Co(II)-C_12_H_18_N_2_O_7_	Co(II)-C_12_H_19_N_2_O_7_	362.0524	362.0528	1.1–4.4	^59^Co	362.0524	100.0		Co	58.9332	58.9330	1.7–8.5
*Metal-NA complexes*													
NA	C_12_H_21_N_3_O_6_	C_12_H_22_N_3_O_6_											
Fe(III)-NA	Fe(III)-C_12_H_18_N_3_O_6_	Fe(III)-C_12_H_19_N_3_O_6_	357.0623	357.0622	0.3–3.9	^56^Fe	357.0623	100.0		*Fe	55.9349	55.9345	3.6–8.9
						^54^Fe	355.0670	6.3	0.7–8.8		53.9396	n.i.	n.i.
						^57^Fe	358.0628	2.4	1.9–5.4		56.9354	n.i.	n.i.
Fe(II)-NA	Fe(II)-C_12_H_19_N_3_O_6_	Fe(II)-C_12_H_20_N_3_O_6_	358.0701	358.0702	0.3–3.4	^56^Fe	358.0701	100.0		Fe	55.9349	55.9344	1.8–8.9
						^54^Fe	356.0748	6.3	0.0–8.9		53.9396	53.9393	1.9–13.0
						^57^Fe	359.0706	2.4	0.3–9.0		56.9354	56.9424	n.a.
Cu(II)-NA	Cu(II)-C_12_H_19_N_3_O_6_	Cu(II)-C_12_H_20_N_3_O_6_	365.0648	365.0652	0.8–3.8	^63^Cu	365.0648	100.0		Cu	62.9296	62.9292	1.6–7.9
						^65^Cu	367.0630	44.6	0.5–5.2		64.9278	64.9274	1.5–9.2
Ni(II)-NA	Ni(II)-C_12_H_19_N_3_O_6_	Ni(II)-C_12_H_20_N_3_O_6_	360.0706	360.0705	0.3–4.2	^58^Ni	360.0706	100.0		Ni	57.9353	57.9350	1.7–6.9
						^60^Ni	362.0660	38.2	0.7–3.8		59.9308	59.9306	3.3–11.7
Zn(II)-NA	Zn(II)-C_12_H_19_N_3_O_6_	Zn(II)-C_12_H_20_N_3_O_6_	366.0644	366.0656	1.6–4.6	^64^Zn	366.0644	100.0		Zn	63.9291	63.9290	0.0–4.7
						^66^Zn	368.0612	57.4	2.5–8.3		65.9260	62.9257	4.6–12.1
						^68^Zn	370.0601	38.7	1.1–9.2		67.9248	67.9246	2.9–10.3
Co(II)-NA	Co(II)-C_12_H_19_N_3_O_6_	Co(II)-C_12_H_20_N_3_O_6_	361.0684	361.0682	0.6–5.0	^59^Co	361.0684	100.0		Co	58.9332	58.9328	1.7–8.5

Data were obtained in positive ESI mode with Orbitrap-MS. Mass accuracy of metal–DMA/NA complexes in MS1 and their released free metals in MS2 are quoted in parts per million (ppm, see Supplementary Tables S1 and S2), n=8. Isotopic mass accuracy was estimated with the calculated errors in relative isotopic abundance (RIA, see Supplementary Table S3), n=8. [M] and [M+H]^+^ indicate the neutral molecule and the protonated ions, respectively. Asterisk denotes Fe isotope released from Fe(III)–NA complex. n.a.-not accurate; n.i.-not identified.

**Table 3:** Relative abundance of the released free Cu and Ni isotopes from Cu(II)/Ni(II)–NA complexes.

Relative abundance of ^63^Cu released from ^63^Cu(II)-NA, *m/z* 365 at different HCD						
*Cu(II)-NA std sol.*	*40*	*50*	*60*	*70*	*80*	*90*
1x	0.025	0.268	0.426	3.531	4.912	9.818
dilution 1:2	0.021	0.209	0.256	1.567	3.406	5.945
dilution 1:4	0.010	0.099	0.328	0.925	2.053	3.751
dilution 1:6		0.087	0.225	0.690	1.390	2.417
dilution 1:8		0.056	0.191	0.518	1.027	1.937
dilution 1:10		0.054	0.148	0.439	0.860	1.443
Relative abundance of ^65^Cu released from ^65^Cu(II)-NA, *m/z* 367 at different HCD						
*Cu(II)-NA std sol.*	*40*	*50*	*60*	*70*	*80*	*90*
1x	0.004	0.079	0.373	1.046	2.053	2.257
dilution 1:2		0.070	0.237	0.419	1.019	1.824
dilution 1:4		0.037	0.133	0.372	0.705	1.280
dilution 1:6		0.026	0.105	0.228	0.480	0.777
dilution 1:8		0.015	0.081	0.189	0.396	0.593
dilution 1:10		0.017	0.056	0.146	0.295	0.530
Relative abundance of ^58^Ni released from ^58^Ni(II)-NA, *m/z* 360 at different HCD						
*Ni(II)-NA std sol.*	*40*	*50*	*60*	*70*	*80*	*90*
1x			0.009	0.044	0.156	0.254
dilution 1:2			0.004	0.046	0.114	0.248
dilution 1:4				0.048	0.111	0.207
dilution 1:6				0.034	0.103	0.176
dilution 1:8				0.028	0.080	0.174
dilution 1:10				0.021	0.062	0.135
Relative abundance of ^60^Ni released from ^60^Ni(II)-NA, *m/z* 362 at different HCD						
*Ni(II)-NA std sol.*	*40*	*50*	*60*	*70*	*80*	*90*
1x				0.027	0.049	0.127
dilution 1:2				0.023	0.071	0.103
dilution 1:4				0.023	0.057	0.095
dilution 1:6				0.021	0.047	0.095
dilution 1:8				0.021	0.036	0.090
dilution 1:10				0.016	0.034	0.089

MS/MS data were obtained in positive ESI mode in Orbitrap-MS at different percentages of high-energy collisional dissociation (HCD: 40–90% shown in italics). The relative abundance of the released free metals was obtained by normalizing the peak intensity of the free metals, *m/z* 62.929 and 64.927 for ^63^Cu and ^65^Cu, respectively; *m/z* 57.935 and 59.930 for ^58^Ni and ^60^Ni, respectively, to that of the internal standard (leucine enkephalin, 2.0 ppm). Metal–NA standard complex solutions (*std sol.*) were prepared in distilled deionized water and further diluted up to 10-fold (dilution 1x to 1:10).
