# Supplementary material for: Identification of metal species by ESI-MS/MS through release of free metals from the corresponding metal-ligand complexes
Source: Sci Rep. 2016 May 31;6:26785. doi: 10.1038/srep26785 (PMC4886218; doi:10.1038/srep26785)
Supplement: Supplementary Information [file srep26785-s1.pdf]

Identification of metal species by ESI-MS/MS through release of free metals from the corresponding metal-ligand complexes

Munkhtsetseg Tsednee, Yu-Chen Huang, Yet-Ran Chen and Kuo-Chen Yeh

Table S1. Estimation of calculated and observed *m/z* of metal-DMA/NA complexes

	Elemental composition			No1	No2	No3	No4	No5	No6	No7	No8	
	[M]	$[M+H]^+$	calculated *m/z*	observed *m/z*	observed *m/z*	observed *m/z*	observed *m/z*	observed *m/z*	observed *m/z*	observed *m/z*	observed *m/z*	
DMA	$C_{12}H_{20}N_2O_7$	$C_{12}H_{21}N_2O_7$										
Fe(III)-DMA	Fe(III)-$C_{12}H_{17}N_2O_7$	Fe(III)-$C_{12}H_{18}N_2O_7$	358.0463	358.0464	358.0467	358.0463	358.0463	358.0466	358.0458	358.0456	358.0461	
Cu(II)-DMA	Cu(II)-$C_{12}H_{18}N_2O_7$	Cu(II)-$C_{12}H_{19}N_2O_7$	366.0488	366.0480	366.0493	366.0486	366.0484	366.0483	366.0480	366.0495	366.0500	
Ni(II)-DMA	Ni(II)-$C_{12}H_{18}N_2O_7$	Ni(II)-$C_{12}H_{19}N_2O_7$	361.0546	361.0548	361.0544	361.0545	361.0547	361.0545	361.0540	361.0554	361.0557	
Zn(II)-DMA	Zn(II)-$C_{12}H_{18}N_2O_7$	Zn(II)-$C_{12}H_{19}N_2O_7$	367.0484	367.0494	367.0476	367.0501	367.0479	367.0495	367.0502	367.0495	367.0502	
Co(II)-DMA	Co(II)-$C_{12}H_{18}N_2O_7$	Co(II)-$C_{12}H_{19}N_2O_7$	362.0524	362.0528	362.0539	362.0533	362.0529	362.0540	362.0538	362.0538	362.0540	
				Δ ppm	Δ ppm	Δ ppm	Δ ppm	Δ ppm	Δ ppm	Δ ppm	Δ ppm	range of Δ ppm
		Fe(III)-$C_{12}H_{18}N_2O_7$		0.3	1.1	0.0	0.0	0.8	1.4	2.0	0.6	0.0-2.0
		Cu(II)-$C_{12}H_{19}N_2O_7$		2.2	1.4	0.5	1.1	1.4	2.2	1.9	3.3	0.5-3.3
		Ni(II)-$C_{12}H_{19}N_2O_7$		0.6	0.6	0.3	0.3	0.3	1.7	2.2	3.0	0.3-3.0
		Zn(II)-$C_{12}H_{19}N_2O_7$		2.7	2.2	4.6	1.4	3.0	4.9	3.0	4.9	1.4-4.9
		Co(II)-$C_{12}H_{19}N_2O_7$		1.1	4.1	2.5	1.4	4.4	3.9	3.9	4.4	1.1-4.4
			calculated *m/z*	observed *m/z*	observed *m/z*	observed *m/z*	observed *m/z*	observed *m/z*	observed *m/z*	observed *m/z*	observed *m/z*	
NA	$C_{12}H_{21}N_3O_6$	$C_{12}H_{22}N_3O_6$										
Fe(III-NA	Fe(III)-$C_{12}H_{18}N_3O_6$	Fe(III)-$C_{12}H_{19}N_3O_6$	357.0623	357.0622	357.0635	357.0632	357.0637	357.0620	357.0618	357.0626	357.0631	
Fe(II)-NA	Fe(II)-$C_{12}H_{19}N_3O_6$	Fe(II)-$C_{12}H_{20}N_3O_6$	358.0701	358.0702	358.0712	358.0709	358.0713	358.0708	358.0710	358.0712	358.0708	
Cu(II)-NA	Cu(II)-$C_{12}H_{19}N_3O_6$	Cu(II)-$C_{12}H_{20}N_3O_6$	365.0648	365.0652	365.0658	365.0660	365.0656	365.0643	365.0659	365.0651	365.0662	
Ni(II)-NA	Ni(II) -$C_{12}H_{19}N_3O_6$	Ni(II)-$C_{12}H_{20}N_3O_6$	360.0706	360.0705	360.0716	360.0721	360.0713	360.0721	360.0712	360.0708	360.0712	
Zn(II)-NA	Zn(II) -$C_{12}H_{19}N_3O_6$	Zn(II)-$C_{12}H_{20}N_3O_6$	366.0644	366.0656	366.0650	366.0657	366.0656	366.0655	366.0651	366.0661	366.0657	
Co(II)-NA	Co(II)-$C_{12}H_{19}N_3O_6$	Co(II)-$C_{12}H_{20}N_3O_6$	361.0684	361.0682	361.0696	361.0700	361.0686	361.0697	361.0702	361.0698	361.0679	
				Δ ppm	Δ ppm	Δ ppm	Δ ppm	Δ ppm	Δ ppm	Δ ppm	Δ ppm	range of Δ ppm
		Fe(III)-$C_{12}H_{19}N_3O_6$		0.3	3.4	2.5	3.9	0.8	1.4	0.8	2.2	0.3-3.9
		Fe(II)-$C_{12}H_{20}N_3O_6$		0.3	3.1	2.2	3.4	2.0	2.5	3.1	2.0	0.3-3.4
		Cu(II)-$C_{12}H_{20}N_3O_6$		1.1	2.7	3.3	2.2	1.4	3.0	0.8	3.8	0.8-3.8
		Ni(II)-$C_{12}H_{20}N_3O_6$		0.3	2.8	4.2	1.9	4.2	1.7	0.6	1.7	0.3-4.2
		Zn(II)-$C_{12}H_{20}N_3O_6$		3.3	1.6	3.6	3.3	3.0	1.9	4.6	3.6	1.6-4.6
		Co(II)-$C_{12}H_{20}N_3O_6$		0.6	3.3	4.4	0.6	3.6	5.0	3.9	1.4	0.6-5.0

Data were obtained in positive ESI mode in Orbitrap-MS with eight replicates (No1-No8) of complex standard samples. Observed *m/z* of metal complexes were noted in 2 ppm mass tolerance window. Mass accuracy is quoted in parts per million (ppm).

Table S2. Estimation of isotopic abundance accuracy from metal-DMA/NA complexes

	Elemental composition $[M+H]^+$	metal isotope	calculated isotopic m/z	calculated isotopic mass intensity, %	No1 observed intensity	No2 observed intensity	No3 observed intensity	No4 observed intensity	No5 observed intensity	No6 observed intensity	No7 observed intensity	No8 observed intensity	
DMA													
Fe(III)-DMA	Fe(III)-$C_{12}H_{18}N_2O_7$	^{56}Fe	358.0463	100.0	7.48E+05	7.35E+05	6.01E+05	3.60E+05	3.79E+05	2.92E+05	2.83E+05	3.75E+05	
		^{54}Fe	356.0510	6.3	4.66E+04	4.45E+04	3.48E+04	2.10E+04	2.10E+04	1.68E+04	1.50E+04	2.09E+04	
		^{57}Fe	359.0468	2.4	1.89E+04	1.70E+04	1.02E+04	1.67E+04	1.78E+04	1.26E+04	4.84E+03	1.93E+04	
Cu(II)-DMA	Cu(II)-$C_{12}H_{19}N_2O_7$	^{63}Cu	366.0488	100.0	1.02E+07	2.19E+07	9.44E+04	8.42E+04	8.41E+04	8.71E+04	2.73E+05	2.94E+05	
		^{65}Cu	368.0470	44.6	4.71E+06	1.01E+07	4.04E+04	3.89E+04	3.70E+04	3.81E+04	1.26E+05	1.39E+05	
Ni(II)-DMA	Ni(II)-$C_{12}H_{19}N_2O_7$	^{58}Ni	361.0546	100.0	1.66E+06	3.14E+04	1.10E+05	1.24E+05	1.16E+05	1.25E+05	6.82E+04	8.23E+04	
		^{60}Ni	363.0500	38.2	6.44E+05	1.27E+04	3.98E+04	4.90E+04	4.39E+04	4.76E+04	2.76E+04	3.06E+04	
Zn(II)-DMA	Zn(II)-$C_{12}H_{19}N_2O_7$	^{64}Zn	367.0484	100.0	4.01E+03	4.13E+03	2.04E+06	1.80E+06	1.03E+04	7.30E+03	1.20E+04	4.77E+03	
		^{66}Zn	369.0453	57.4	2.18E+03	2.30E+03	1.09E+06	9.93E+05	5.80E+03	3.90E+03	7.49E+03	2.50E+03	
		^{68}Zn	371.0441	38.7	1.75E+03	1.91E+03	8.92E+05	6.56E+05	3.50E+03	3.05E+03	5.80E+03	1.40E+03	
Co(II)-DMA	Co(II)-$C_{12}H_{19}N_2O_7$	^{59}Co	362.0524	100.0									
					RIA error, %	RIA error, %	RIA error, %	RIA error, %	RIA error, %	RIA error, %	RIA error, %	RIA error, %	range of RIA error, %
		^{54}Fe			0.1	0.2	0.5	0.5	0.8	0.5	1.0	0.7	0.1-1.0
		^{57}Fe			0.1	0.1	0.7	2.2	2.3	1.9	0.7	2.7	0.1-2.7
		^{65}Cu			1.6	1.5	1.8	1.6	0.6	0.9	1.6	2.7	0.6-2.7
		^{60}Ni			0.6	2.2	2.0	1.3	0.4	0.1	2.3	1.0	0.1-2.3
		^{66}Zn			3.0	1.7	4.0	2.2	1.1	4.0	5.0	5.0	1.1-5.0
		^{68}Zn			4.9	7.5	5.0	2.3	4.7	3.1	9.6	9.3	2.3-9.6
NA					observed intensity	observed intensity	observed intensity	observed intensity	observed intensity	observed intensity	observed intensity	observed intensity	
Fe(III-NA	Fe(III)-$C_{12}H_{19}N_3O_6$	^{56}Fe	357.0623	100.0	2.64E+05	2.47E+05	1.07E+06	1.08E+06	1.45E+04	2.91E+05	4.92E+04	2.33E+04	
		^{54}Fe	355.0670	6.3	3.98E+04	2.61E+04	7.82E+04	7.57E+04	1.26E+03	3.22E+04	5.88E+03	1.74E+03	
		^{57}Fe	358.0628	2.4	1.34E+04	1.93E+04	3.76E+03	2.52E+03	9.99E+02	2.18E+04	2.13E+03	1.09E+03	
Fe(II)-NA	Fe(II)-$C_{12}H_{20}N_3O_6$	^{56}Fe	358.0701	100.0	9.09E+05	7.64E+05	4.79E+05	4.65E+05	9.96E+03	9.08E+05	1.60E+04	1.86E+04	
		^{54}Fe	356.0748	6.3	6.40E+04	5.06E+04	3.00E+04	3.06E+04	1.51E+03	7.03E+04	2.21E+03	2.73E+03	
		^{57}Fe	359.0706	2.4	2.90E+04	2.07E+04	1.86E+04	1.82E+04	4.02E+02	1.63E+04	1.82E+03	1.53E+03	
Cu(II)-NA	Cu(II)-$C_{12}H_{20}N_3O_6$	^{63}Cu	365.0648	100.0	8.89E+06	8.32E+06	7.05E+05	4.28E+06	1.02E+06	4.44E+06	2.17E+06	1.29E+06	
		^{65}Cu	367.0630	44.6	4.07E+06	3.85E+06	2.89E+05	2.13E+06	4.50E+05	2.12E+06	1.04E+06	6.07E+05	
Ni(II)-NA	Ni(II)-$C_{12}H_{20}N_3O_6$	^{58}Ni	360.0706	100.0	2.79E+05	4.69E+05	4.75E+05	4.59E+05	5.47E+05	6.74E+05	5.15E+05	4.72E+05	
		^{60}Ni	362.0660	38.2	1.13E+05	1.97E+05	1.85E+05	1.81E+05	2.14E+05	2.55E+05	2.02E+05	1.99E+05	
Zn(II)-NA	Zn(II)-$C_{12}H_{20}N_3O_6$	^{64}Zn	366.0644	100.0	2.51E+03	3.06E+03	1.94E+06	2.31E+06	4.48E+03	2.11E+05	1.16E+05	4.92E+05	
		^{66}Zn	368.0612	57.4	1.37E+03	2.01E+03	1.01E+06	1.21E+06	2.46E+03	1.32E+05	7.28E+04	3.03E+05	
		^{68}Zn	370.0601	38.7	7.69E+02	1.02E+03	5.96E+05	7.03E+05	1.32E+03	7.81E+04	4.70E+04	1.85E+05	
Co(II)-NA	Co(II)-$C_{12}H_{20}N_3O_6$	^{59}Co	361.0684	100.0									
					RIA error, %	RIA error, %	RIA error, %	RIA error, %	RIA error, %	RIA error, %	RIA error, %	RIA error, %	range of RIA error, %
	Fe(III)-NA	^{54}Fe			8.8	4.3	1.0	0.7	2.4	4.8	5.7	1.2	0.7-8.8
		^{57}Fe			2.7	5.4	2.0	2.2	4.5	5.1	1.9	2.3	1.9-5.4
	Fe(II)-NA	^{54}Fe			0.7	0.3	0.0	0.3	8.9	1.4	7.5	8.4	0.0-8.9
		^{57}Fe			0.8	0.3	1.5	1.5	1.6	0.6	9.0	5.8	0.3-9.0
		^{65}Cu			1.2	1.7	3.6	5.2	0.5	3.1	3.3	2.5	0.5-5.2
		^{60}Ni			2.3	3.8	0.7	1.2	0.9	0.4	1.0	4.0	0.7-3.8
		^{66}Zn			2.8	8.3	5.3	5.0	2.5	5.2	5.4	4.2	2.5-8.3
		^{68}Zn			8.1	5.4	8.0	8.3	9.2	1.7	1.8	1.1	1.1-9.2

Data were obtained in positive ESI mode in Orbitrap-MS with eight replicates (No1-No8) of complex standard samples and mass tolerance window was 2 ppm. Calculated isotopic *m/z* and the calculated mass intensity were obtained from Scientific Instrument Service (http://www.sisweb.com/mstools/isotope.htm). Isotopic abundance accuracy is estimated with the calculated errors in relative isotopic abundance (RIA, %).

Table S3. Estimation of calculated and observed *m/z* of the released free metal from metal-DMA/NA complexes

	Elemental composition [M+H]+	*m/z* of precursor	Released free metal	calculated *m/z*	No1 observed *m/z*	No2 observed *m/z*	No3 observed *m/z*	No4 observed *m/z*	No5 observed *m/z*	No6 observed *m/z*	No7 observed *m/z*	No8 observed *m/z*	
DMA													
Fe(III)-DMA	Fe(III)-$C_{12}H_{18}N_2O_7$	358.0463	Fe	55.9349	55.9344	55.9346	55.9344	55.9344	55.9346	55.9347	55.9347	55.9344	
		356.0510		53.9389	53.9391	53.9388	53.9393	53.939	53.9389	53.9388	53.9393	53.9389	
		359.0468		56.9354	56.9420	56.9422	56.9426	56.9423	56.9424	56.9420	56.9424	56.9422	
Cu(II)-DMA	Cu(II)-$C_{12}H_{19}N_2O_7$	366.0488	Cu	62.9296	62.9294	62.9292	62.9292	62.9295	62.9291	62.9293	62.9293	62.9290	
		368.0470		64.9278	64.9274	64.9276	64.9274	64.9274	64.9281	64.9277	64.9274	64.9273	
Ni(II)-DMA	Ni(II)-$C_{12}H_{19}N_2O_7$	361.0546	Ni	57.9353	57.9352	57.9352	57.9351	57.9351	57.9349	57.9352	57.9350	57.9351	
		363.0500		59.9308	59.9304	59.9304	59.9303	59.9306	59.9304	59.9310	59.9301	59.9304	
Zn(II)-DMA	Zn(II)-$C_{12}H_{19}N_2O_7$	367.0484	Zn	63.9291	63.9290	63.9294	63.9297	63.9292	63.9290	63.9296	63.9296	63.9297	
				65.9260	65.9256	65.9259	65.9253	65.9259	65.9251	65.9252	65.9256	65.9253	
				67.9248	67.9244	67.9244	67.9241	67.9242	67.9240	67.9244	67.9241	67.9245	
Co(II)-DMA	Co(II)-$C_{12}H_{19}N_2O_7$	362.0524	Co	58.9332	58.9330	58.9330	58.9327	58.9328	58.9327	58.9329	58.9331	58.9328	
					Δ ppm	Δ ppm	Δ ppm	Δ ppm	Δ ppm	Δ ppm	Δ ppm	Δ ppm	range of Δ ppm
			Fe		8.9	5.4	8.9	8.9	5.4	3.6	3.6	8.9	3.6-8.9
					13.0	9.3	5.6	13.0	11.1	13.0	5.6	9.3	5.6-13.0
					n.a.	n.a.	n.a.	n.a.	n.a.	n.a.	n.a.	n.a.	n.a.
			Cu		3.2	6.4	6.4	1.6	7.9	4.8	4.8	9.5	1.6-9.5
					6.2	3.1	6.2	6.2	4.6	1.5	6.2	7.7	1.5-7.7
			Ni		1.7	1.7	3.5	3.5	6.9	1.7	5.2	3.5	1.7-6.9
					6.7	6.7	8.3	3.3	6.7	3.3	11.7	6.7	3.3-11.7
			Zn		1.6	4.7	9.4	1.6	1.6	7.8	7.8	9.4	1.6-9.4
					6.1	1.5	10.6	1.5	13.7	12.1	6.1	10.6	1.5-13.7
					5.9	5.9	10.3	8.8	11.8	5.9	10.3	4.4	4.4-10.3
			Co		3.4	3.4	8.5	6.8	8.5	5.1	1.7	6.8	1.7-8.5
				calculated *m/z*	observed *m/z*	observed *m/z*	observed *m/z*	observed *m/z*	observed *m/z*	observed *m/z*	observed *m/z*	observed *m/z*	
NA													
Fe(III-NA	Fe(III)-$C_{12}H_{19}N_3O_6$	357.0623	Fe*	55.9349	55.9345	55.9344	55.9346	55.9344	55.9346	55.9347	55.9347	55.9346	
Fe(II)-NA	Fe(II)-$C_{12}H_{20}N_3O_6$	358.0701	Fe	55.9349	55.9344	55.9348	55.9347	55.9345	55.9347	55.9346	55.9346	55.9347	
		356.0748		53.9396	53.9393	53.9395	53.9391	53.9389	53.9391	53.9397	53.9392	53.9389	
		359.0706		56.9354	56.9424	56.9426	56.9424	56.9423	56.9424	56.9422	56.9424	56.9241	
Cu(II)-NA	Cu(II)-$C_{12}H_{20}N_3O_6$	365.0648	Cu	62.9296	62.9292	62.9292	62.9292	62.9291	62.9294	62.9295	62.9298	62.9292	
		367.0630		64.9278	64.9274	64.9274	64.9276	64.9274	64.9277	64.9274	64.9272	64.9273	
Ni(II)-NA	Ni(II)-$C_{12}H_{20}N_3O_6$	360.0706	Ni	57.9353	57.9350	57.9352	57.9350	57.9352	57.9351	57.9349	57.9350	57.9352	
		362.0660		59.9308	59.9306	59.9304	59.9304	59.9303	59.9304	59.9306	59.9304	59.9301	
Zn(II)-NA	Zn(II)-$C_{12}H_{20}N_3O_6$	366.0644	Zn	63.9291	63.9290	63.9289	63.9294	63.9291	63.9294	63.9290	63.9288	63.9289	
				65.9260	65.9252	65.9254	65.9252	65.9252	65.9256	65.9257	65.9256	65.9252	
				67.9248	67.9242	67.9241	67.9241	67.9241	67.9240	67.9246	67.9245	67.9242	
Co(II)-NA	Co(II)-$C_{12}H_{20}N_3O_6$	361.0684	Co	58.9332	58.9328	58.9329	58.9327	58.9327	58.9330	58.9331	58.9327	58.9331	
					Δ ppm	Δ ppm	Δ ppm	Δ ppm	Δ ppm	Δ ppm	Δ ppm	Δ ppm	range of Δ ppm
			Fe*		7.2	8.9	5.4	8.9	5.4	3.6	3.6	5.4	3.6-8.9
			Fe		8.9	1.8	3.6	7.2	3.6	5.4	5.4	3.6	1.8-8.9
					5.6	1.9	9.3	13.0	9.3	1.9	7.4	13.0	1.9-13.0
					n.a.	n.a.	n.a.	n.a.	n.a.	n.a.	n.a.	n.a.	n.a.
			Cu		6.4	6.4	6.4	7.9	3.2	1.6	3.2	6.4	1.6-7.9
					6.2	6.2	3.1	6.2	1.5	6.2	9.2	7.7	1.5-9.2
			Ni		5.2	1.7	5.2	1.7	3.5	6.9	5.2	1.7	1.7-6.9
					3.3	6.7	6.7	8.3	6.7	3.3	6.7	11.7	3.3-11.7
			Zn		1.6	3.1	4.7	0.0	4.7	1.6	4.7	3.1	0.0-4.7
					12.1	9.1	12.1	12.1	6.1	4.6	6.1	12.1	4.6-12.1
					8.8	10.3	10.3	10.3	11.8	2.9	4.4	8.8	2.9-10.3
			Co		6.8	5.1	8.5	8.5	3.4	1.7	8.5	1.7	1.7-8.5

Data were obtained in positive ESI mode in Orbitrap-MS with eight replicates (No1-No8) of complex standard samples. Observed *m/z* of metal complexes were noted in 2 ppm mass tolerance window and fragmentation energies of HCD:70-90% for Fe/Cu/Ni/Co-complexes and 120-150% for Zn-complexes in MS/MS. Mass accuracy is quoted in parts per million (ppm). Asterisk denotes Fe isotope released from Fe(III)-NA complex. n.a.-not accurate.

Table S4. Interpretation of neutral lost during fragmentation of metal-DMA/NA complexes

m/z of neutral lost	interpretation of neutral lost
28	CHNH
43	CH_2CHNH_2
44	$CH_2CH_2NH_2$
44	COO
45	COOH
46	HCOOH
54	CHCHCHNH
56	$CH_2CHCHNH_2$
57	$CH_2CH_2CH_2NH_2$
58	$CH_3CH_2CH_2NH_2$

Neutral lost products were proposed from the fragmentations of metal-DMA and metal-NA complexes (Figure 3 and Supplementary Figure S3-S5).

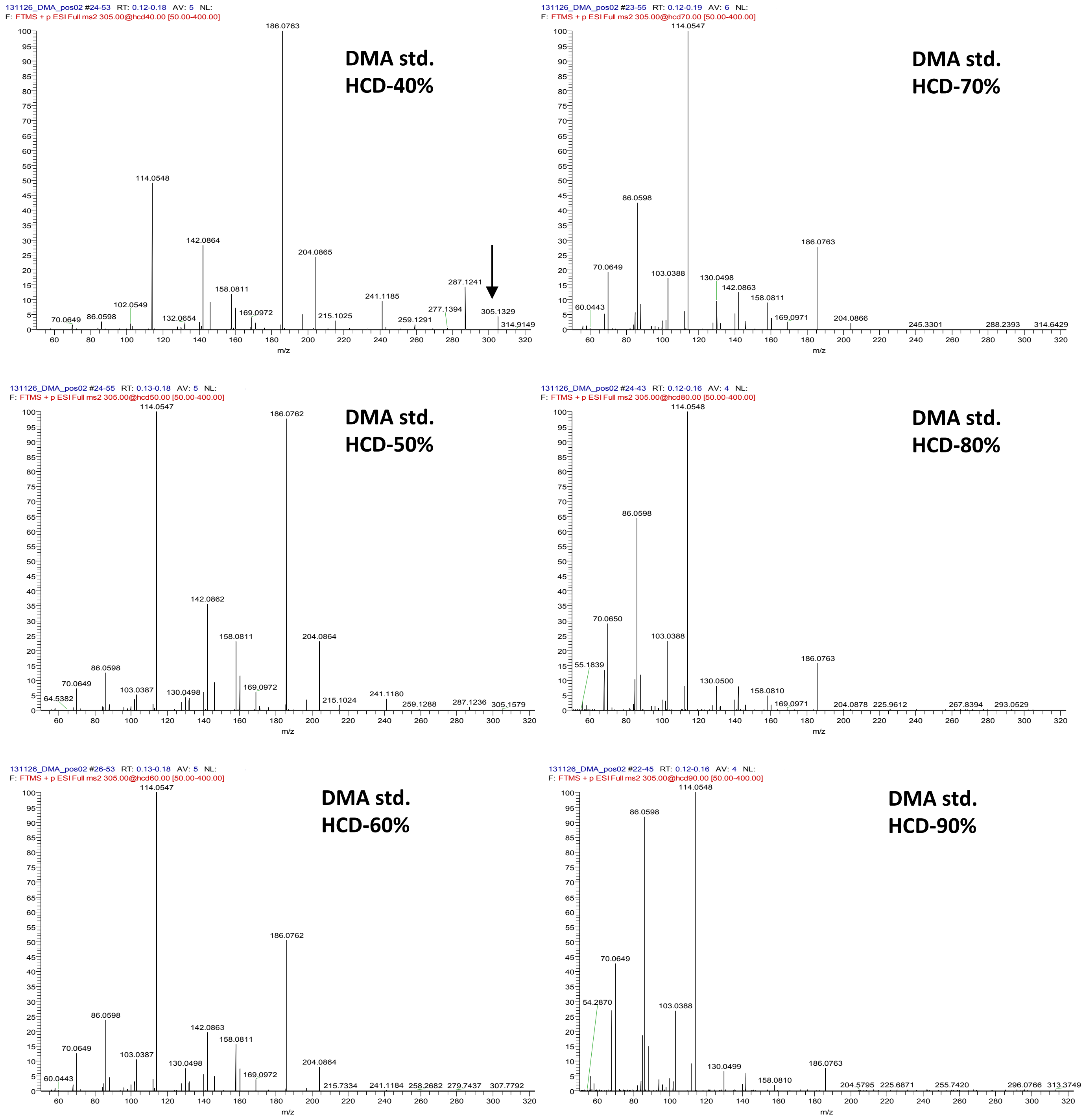

Figure S1. MS/MS spectra of DMA standard. Mass product ions of free DMA standard (std.) were obtained in positive ESI mode with the presented different collision energies (HCD, 40-90%) in Orbitrap-MS. Arrow indicates the spectrum of the precursor ion.

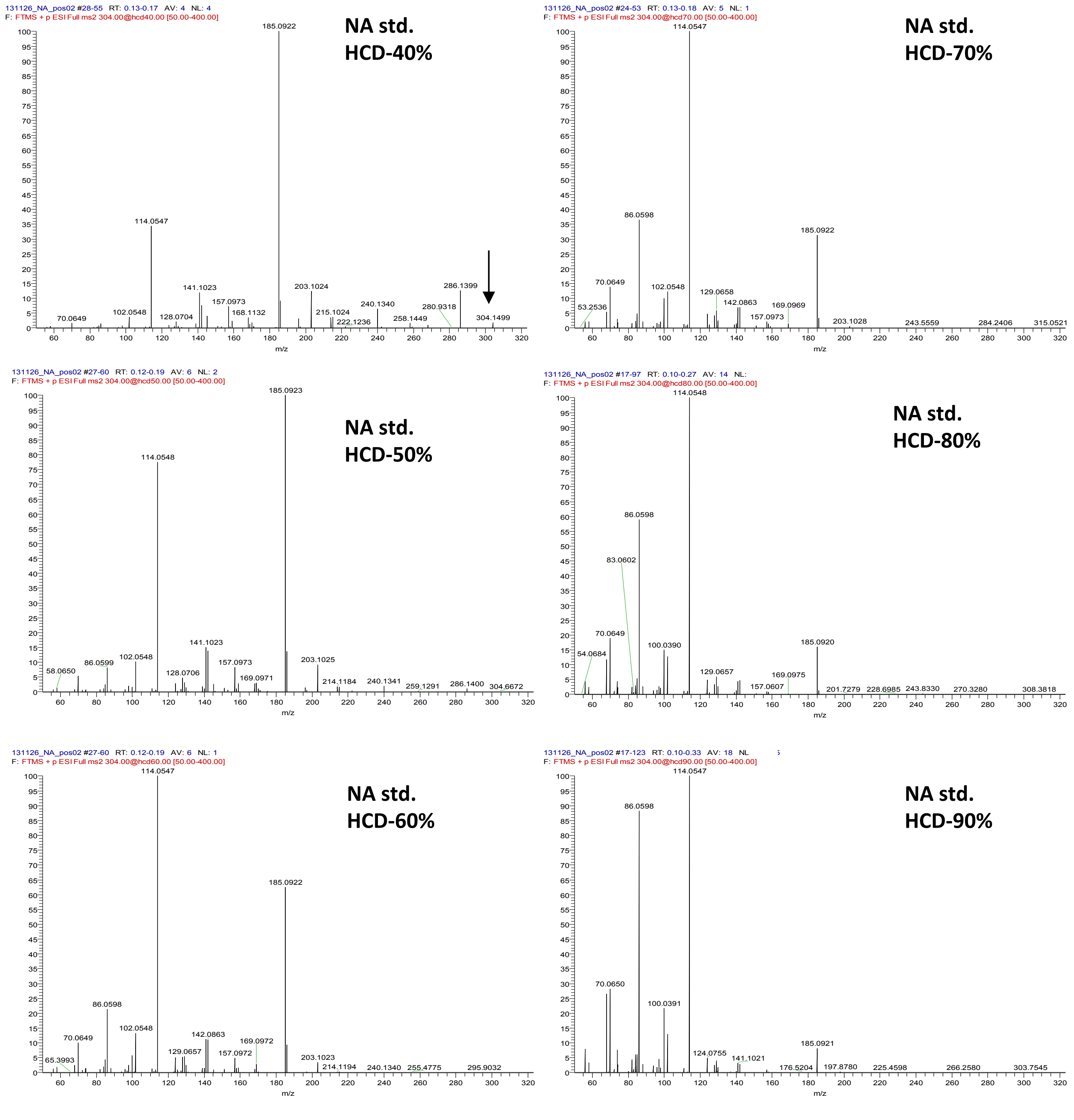

Figure S2. MS/MS spectra of NA standard. Mass product ions of free NA standard (std.) were obtained in positive ESI mode with the presented different collision energies (HCD, 40-90%) in Orbitrap-MS. Arrow indicates the spectrum of the precursor ion.

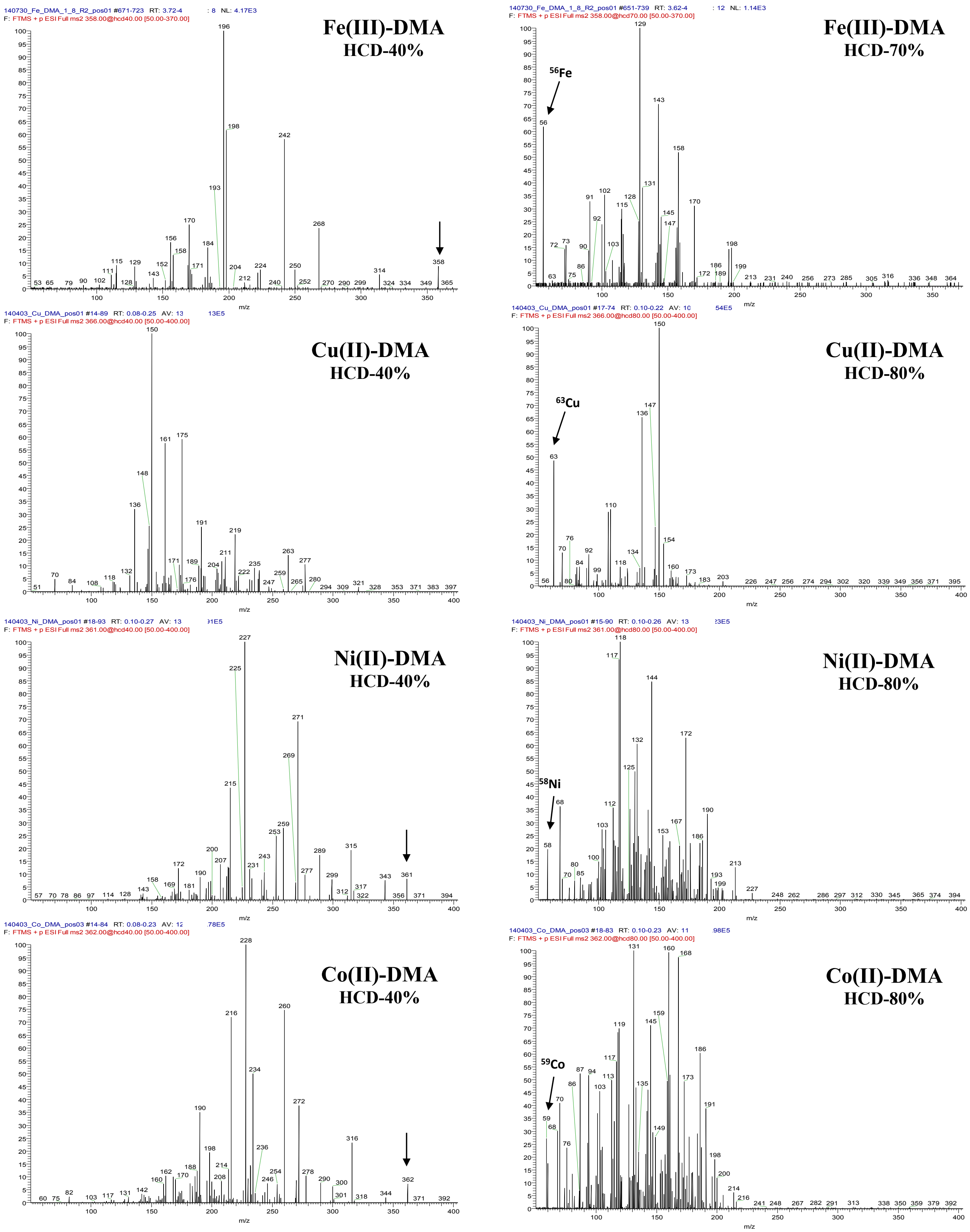

Figure S3. MS/MS spectra of metal-DMA complexes. In ESI-MS/MS, the product ions were obtained at presented collision energies (HCD) using positive ESI mode in Orbitrap-MS instrument. Arrows indicate the spectra of the precursor metal complex ions and the spectra of the released free metals were also marked.

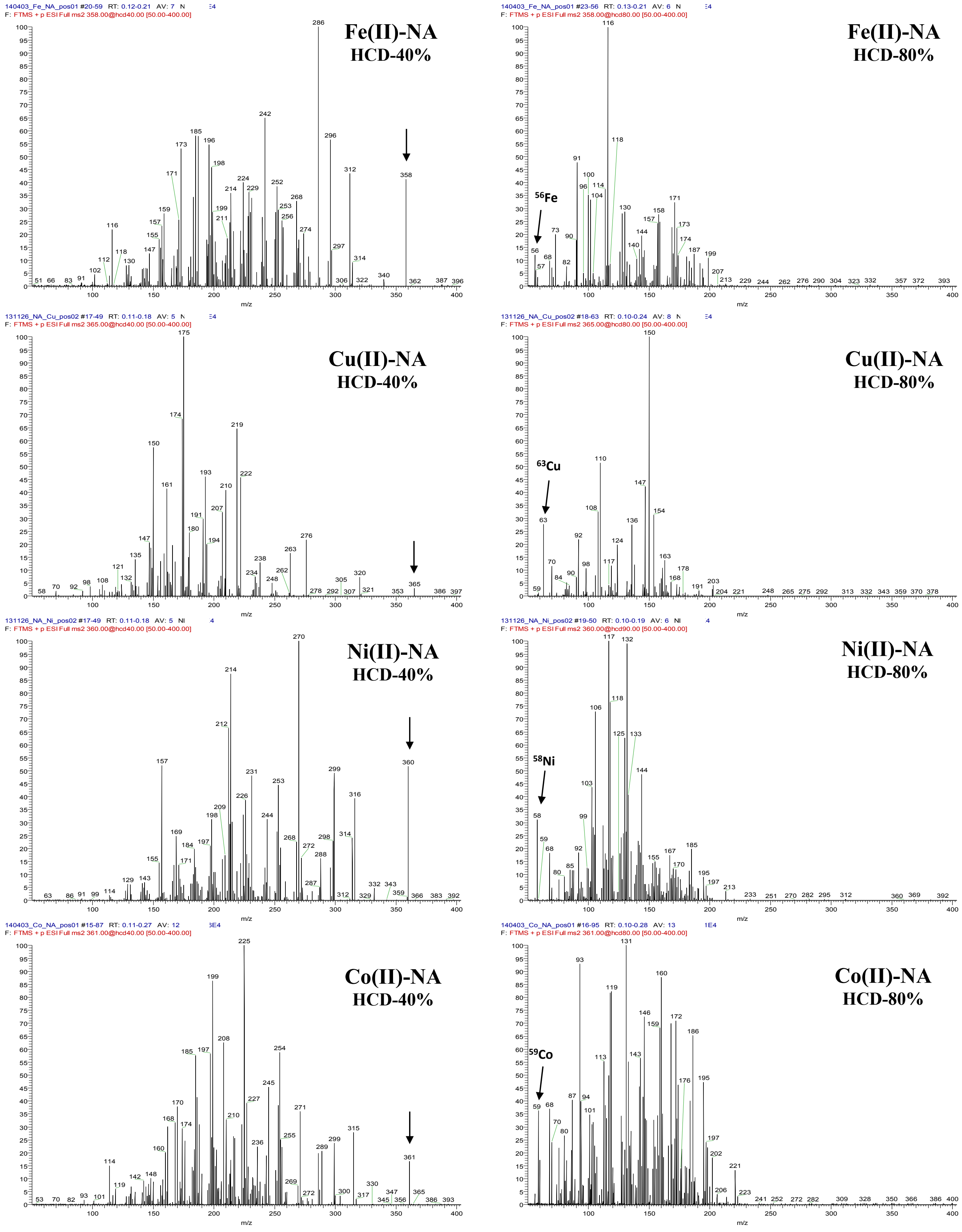

Figure S4. MS/MS spectra of metal-NA complexes. The product ions in ESI-MS/MS were obtained at presented collision energies (HCD) using positive ESI mode in Orbitrap-MS instrument. Arrows indicate the spectra of the precursor metal complex ions and the spectra of the released free metals were also marked.

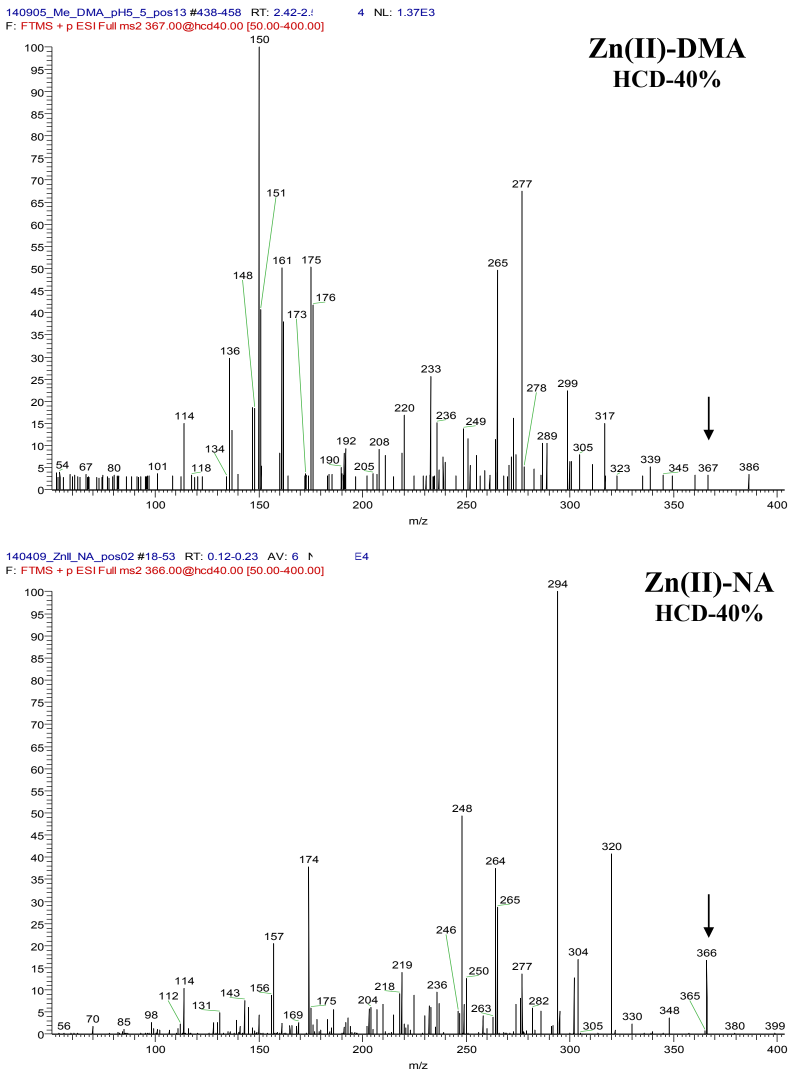

Figure S5. MS/MS spectra of Zn(II)-DMA and Zn(II)-NA complexes. The product ions in ESI-MS/MS were obtained at fragmentation energy of HCD-40% and positive ESI mode in Orbitrap-MS. Arrows indicate the spectra of the precursor metal complex ions.

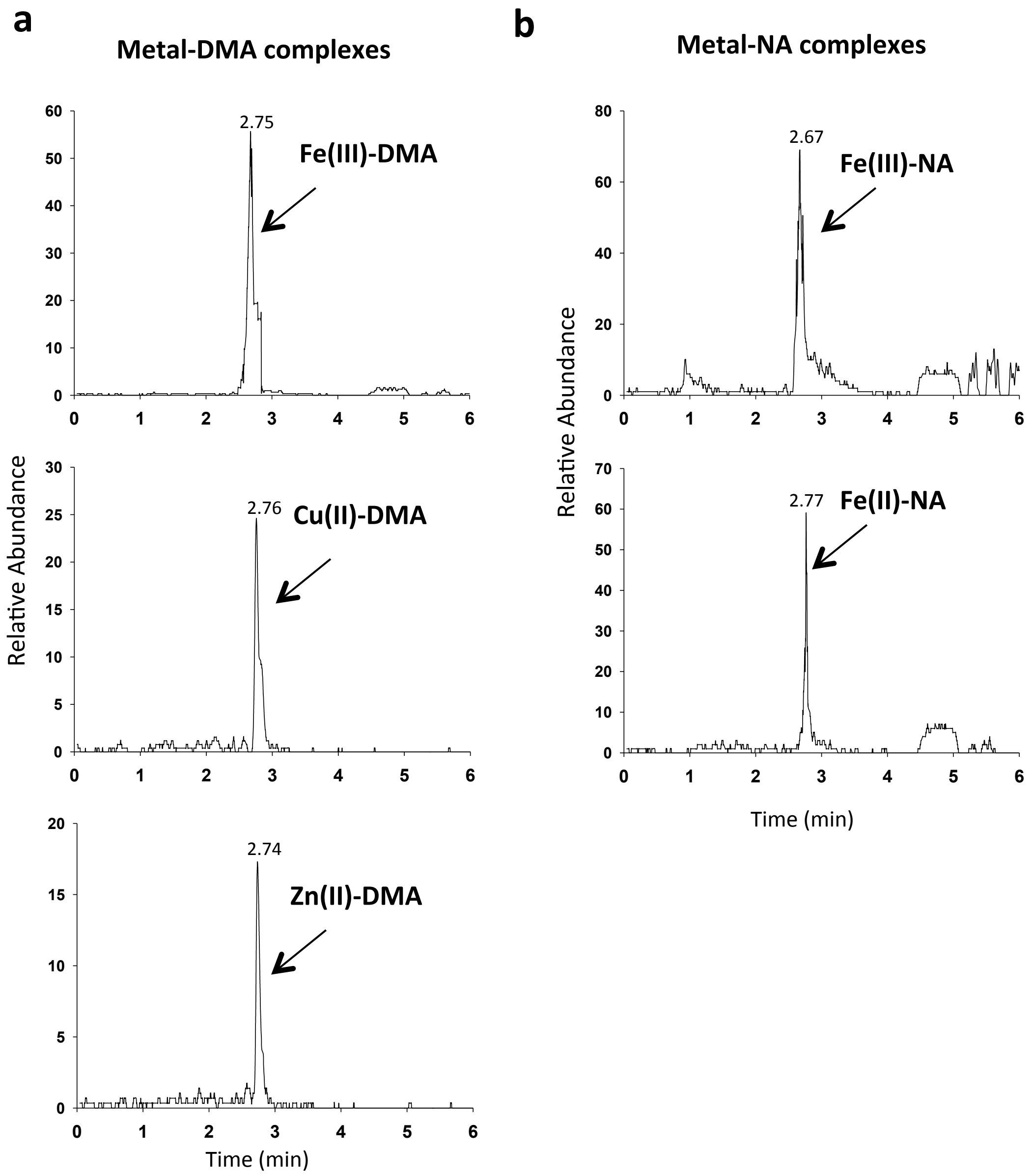

Figure S6. Chromatograms of the identified metal-DMA and metal-NA complexes in shoots of indica rice. Metal-DMA (a) and metal-NA (b) complexes in shoot extracts of rice were identified in positive ESI mode with hydrophilic HILIC column separation in UPLC-ESI-Q-TOF-MS.

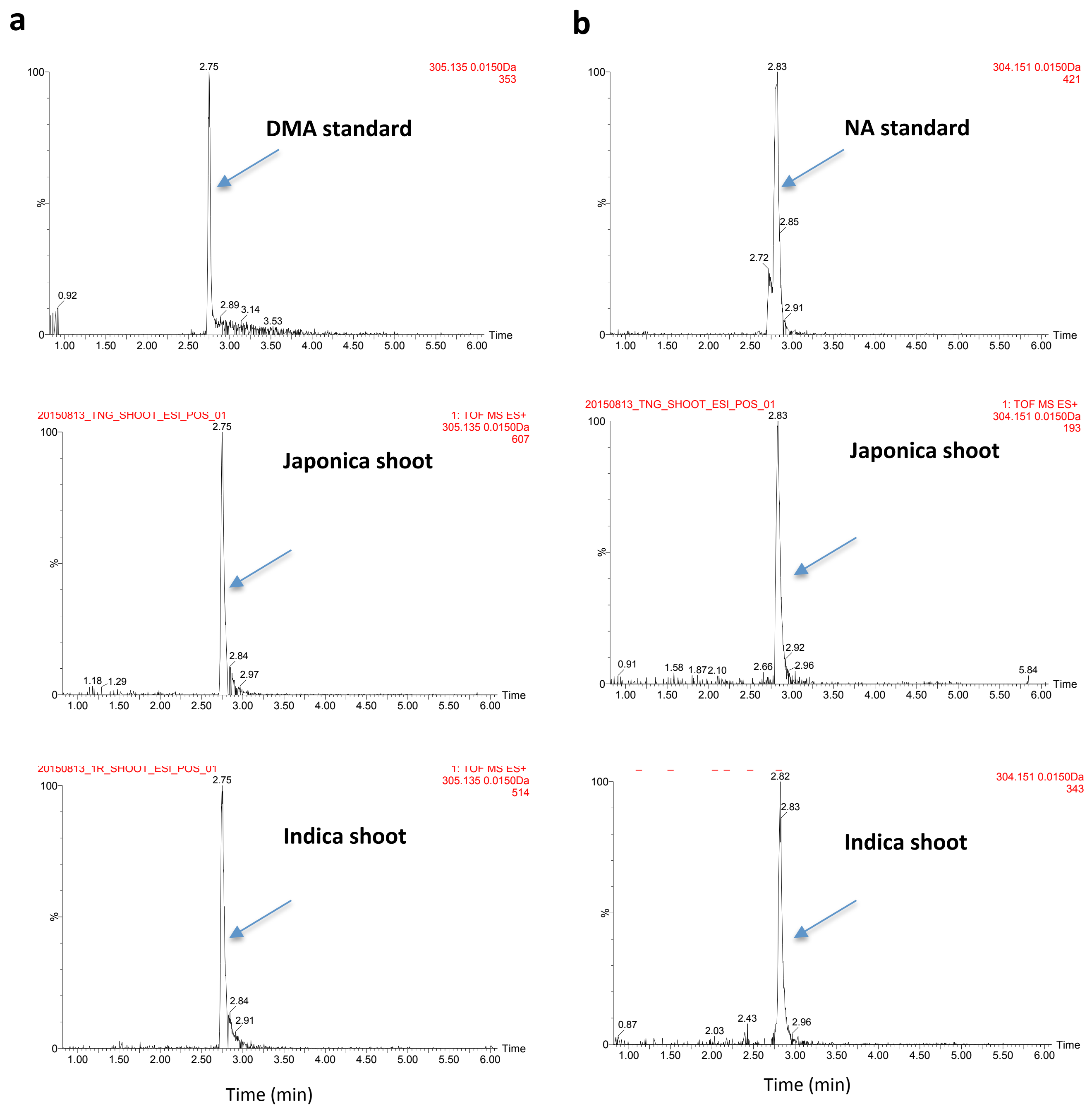

Figure S7. Identification of DMA and NA in rice shoots. DMA (a) and NA (b) in shoot extracts of Japonica rice and Indica rice were identified in positive ESI mode with hydrophilic HILIC column separation in UPLC-ESI-Q-TOF-MS using DMA and NA standards. Arrows indicate the DMA and NA peaks in (a) and (b), respectively.
